# Hand Dysfunction After Intervention via Distal Versus Conventional Transradial Access: A Meta‐Analysis of Randomized Trials

**DOI:** 10.1002/clc.70350

**Published:** 2026-05-12

**Authors:** Xinyu Fan, Tong Zhou, Feng Li, Ganwei Shi, Chenlei Gu, Ziyang Chen, Gaojun Cai

**Affiliations:** ^1^ Changzhou Wujin People's Hospital, Changzhou Medical Center Nanjing Medical University Changzhou Jiangsu Province China; ^2^ Department of Cardiology Wujin hospital affiliated with Jiangsu University, the Wujin Clinical College of Xuzhou Medical University Changzhou Jiangsu Province China

**Keywords:** distal transradial access, hand dysfunction, randomized trials, transradial access

## Abstract

**Background:**

Percutaneous coronary intervention (PCI) is an important treatment for coronary artery disease (CAD). Distal transradial access (dTRA) has gained popularity as an alternative to conventional transradial access (TRA) in cardiovascular interventional procedures. However, some patients may experience hand dysfunction after undergoing TRA or dTRA procedures.

**Methods:**

In this study, the literature from the PubMed and Web of Science (WOS) databases was reviewed and 11 randomized controlled trials (RCTs) were selected. Meta‐analysis and narrative synthesis were used to evaluate the hand dysfunction and vascular complications.

**Result:**

A total of 6903 participants were included in the study. The average age was 62.8 years, and 66.9% were male. In terms of hand motor dysfunction, pooled data from two trials showed that the risk of dTRA may be lower than that of TRA. In terms of hand sensory dysfunction, a pooled analysis of two studies showed that the incidence of numbness within 24 h postoperatively may be higher with dTRA than with TRA, but the incidence of persistent pain may be lower with dTRA than with TRA. Only one included study reported the incidence of nerve injury. Additionally, a pooled analysis of ten trials demonstrated that dTRA had a lower risk of radial artery occlusion (RAO) than TRA.

**Conclusion:**

Compared with TRA, dTRA may be associated with a lower risk of hand clumsiness, persistent pain, RAO, and AVF, but a potentially higher incidence of hand numbness.

## Graphical abstract

This meta‐analysis of randomized controlled trials found that, compared with the conventional transradial access (TRA), the distal transradial access (dTRA) may reduce hand clumsiness and persistent pain, but it increases hand numbness.

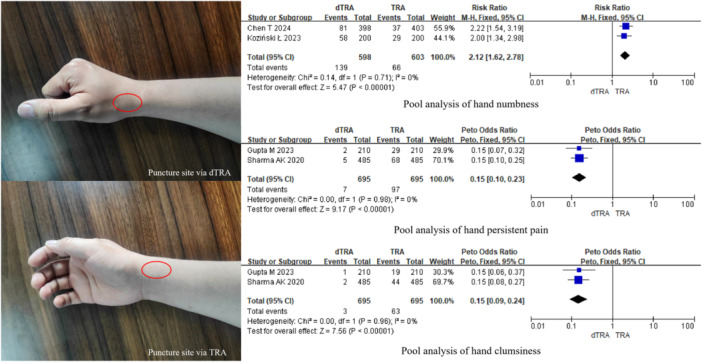

**Abbreviations**
APL: abductor pollicis longus
AS: anatomic snuffbox
AVF: arteriovenous fistula
CAD: coronary artery disease
CI: confidence interval
CV: cephalic vein
DRA: distal radial artery
dTRA: distal transradial access
EPB: extensor pollicis brevis
EPL: extensor pollicis longus
MD: mean difference
mEASY: modified early discharge after transradial stenting of coronary arteries study
NNT: number needed to treat
NNTB: number needed to treat for an additionally beneficial outcome
NNTH: number needed to treat for an additionally harmful outcome
PCI: percutaneous coronary intervention
PRISMA: preferred reporting items for systematic reviews and meta‐analyses
RAO: radial artery occlusion
RCT: randomized controlled trial
RR: risk ratio
SBRN: superficial branch of the radial nerve
TFA: transfemoral access
TRA: transradial artery access

## 1 Background

Coronary artery disease (CAD) is one of the most common cardiovascular diseases and has been identified as the leading cause of death in both developing and developed countries [1], and percutaneous coronary intervention (PCI) is an important treatment for CAD [2]. Coronary intervention requires good vascular access. Compared with transfemoral access (TFA), conventional transradial artery access (TRA) effectively reduces the risk of major bleeding and vascular complications [3]. TRA is recommended as the default access for coronary intervention in the 2018 European Society of Cardiology Guidelines for Myocardial Revascularization, the 2020 Consensus Statement on Best Practices for Transradial Angiography and Intervention, and the 2021 American Heart Association Guidelines for Coronary Revascularization [4, 5, 6].

However, with increased awareness of TRA, a greater incidence of complications, such as radial artery occlusion (RAO), hematoma, pseudoaneurysm, arteriovenous fistula (AVF), and compartmentalized fascial syndrome, has been reported after TRA. In particular, RAO has a high incidence in the early postoperative period, with a meta‐analysis showing a 7.7% incidence of RAO within 24 h after TRA [7]. The occurrence of RAO limits future cardiovascular interventions through the same vascular access and reduces the potential vascular availability for coronary artery bypass grafting and the creation of AVFs in patients requiring hemodialysis. Distal transradial access (dTRA) has emerged as an alternative access to TRA for cardiovascular interventions, because it can significantly reduce RAO, shorten the compression time, and improve patient discomfort [8, 9, 10].

The human hand is naturally equipped to perform labor and enhance productivity. As one of the most important structures of the human body, the function and appearance of the hands are critical. The hand not only provides important support for the upper limb, which is capable of performing a variety of large‐scale movements, but also performs fine manipulations that are closely related to daily life, including grasping, pinching, and finger‐pairing [11]. However, several patients experience abnormal hand sensation as well as symptoms of clumsiness of movement after dTRA or TRA, and the presence of these symptoms may cause some degree of adverse effects [12]. Endothelial thickening, endothelial dysfunction, and nerve injury after vascular puncture may lead to hand dysfunction [13].

## 2 Methods

### 2.1 Study Design

The aim of this study was to review the relevant literature to assess the incidence and clinical symptoms of hand dysfunction and vascular complications after dTRA and TRA by meta‐analysis and narrative integration. The study has been registered in PROSPERO (**ID: CRD420250643415**).

### 2.2 Data Sources and Search Strategies

A systematic review of the PubMed and Web of Science (WOS) databases was performed using the following keywords: (radial OR transradial OR TRA OR radial artery OR distal radial artery OR distal transradial OR distal radial OR DRA OR dTRA OR snuffbox) AND (catheterization OR catheterization OR angiography OR angiogram OR angioplasty OR percutaneous coronary intervention OR PCI) AND (hand function OR grip OR pinch OR disability OR dysfunction OR sensation OR paresthesia OR paralysis) (from inception to May 2025). The references of the retrieved articles were manually searched to increase the recall rate.

### 2.3 Study Screening and Assessment

This study began with a search for any studies assessing hand function after TRA or dTRA procedures. The search results were reviewed by a researcher (XYF), studies were restricted to randomized controlled trials (RCTs), and the language of the study was restricted to English. Hand dysfunction should include hand pain, nerve damage, and motor or sensory abnormalities. There were no restrictions on how hand function was assessed. Data on vascular complications, including vascular occlusion, AVF, pseudoaneurysms, arterial dissection, and hematomas, were collected when reported.

### 2.4 Data Extraction and Analysis

The primary endpoints were indicators related to hand dysfunction, including the incidence of postoperative hand pain, nerve damage, and motor or sensory abnormalities, as well as changes in pinch strength, grip strength, monofilament test results, or hand function questionnaire results. For clarity in terminology, hand dysfunction was categorized into sensory and motor domains. Hand sensory dysfunction refers to abnormal sensations, including numbness and pain, that may impair hand function. Hand motor dysfunction refers to impaired motor performance affecting daily activities. In this study, hand numbness and persistent pain were used as indicators of sensory dysfunction, whereas hand clumsiness, defined as difficulty in performing fine motor tasks, was used as the indicator to assess hand motor dysfunction in the included trials. The secondary endpoints included vascular complications such as any type of local hematoma and bleeding, RAO, AVF, pseudoaneurysm, arterial dissection, arterial spasm, and other vascular complications. Other outcomes, such as the puncture success rate, single puncture success rate, puncture time, procedure time, contrast dosage, and postoperative compression time, were reported if mentioned in the included articles. Data from each study were extracted into *Microsoft Excel* and *Review Manager* (version 5.3) software, and meta‐analysis or pooled analysis was performed for studies with similar interventions. If meta‐analysis or pooled analysis was not possible, a comprehensive narrative was used to report the study results. Partial visualization was achieved through *R* software (version 4.3.1).

The data analyzed in this study included dichotomous and continuous variables. Relative risk ratio (RR) and mean difference (MD) were chosen for dichotomous and continuous variables, respectively, and their 95% confidence interval (CI) was calculated. If continuous variables showed median (upper quartile – lower quartile), their data distribution was tested for significant skewness by statistical methods. If not, they were converted to the mean (standard deviation). Otherwise, the meta‐analysis was discarded, and only a narrative discussion was conducted [14]. For primary outcomes that were amenable to meta‐analysis, the number needed to treat (NNT) was calculated. On the basis of whether dTRA reduces complication rates compared with TRA, NNT was categorized into the number needed to treat for an additionally beneficial outcome (NNTB) and the number needed to treat for an additionally harmful outcome (NNTH).

For dichotomous outcomes, the statistical approach was determined by event occurrence within each study. The Peto method was used as the primary analysis for outcomes with extremely low event rates (sparse data), whereas the Mantel–Haenszel method was used as the primary analysis for all other dichotomous outcomes. Additionally, Mantel–Haenszel models were employed as sensitivity analyses for rare‐event outcomes to assess result robustness.

Studies with zero events in both arms (double‐zero‐event studies) were excluded from meta‐analysis for that specific outcome, as they provide no information for estimating treatment effects, and continuity correction cannot yield valid risk ratios. For outcomes with zero events in only one arm (single‐zero‐event studies), continuity correction was applied to calculate approximate risk ratios and include these studies in the meta‐analysis.

Heterogeneity was assessed by the *I* ^2^ statistic. If *p* ≥ 0.1 and *I* ^2^ < 50%, the heterogeneity among the included studies was acceptable, and meta‐analysis was performed using a fixed‐effects model. If *p* < 0.1 and *I* ^2^ ≥ 50%, heterogeneity was indicated, and sensitivity analyses were required to observe the stability and reliability of the results. If heterogeneity was still not possible, a randomized model was used [15]. Funnel plots were used to assess publication bias. Funnel plots were plotted only for outcomes with 10 or more included studies. For outcomes with fewer than 10 studies, funnel plots were not plotted because insufficient statistical power made it impossible to reliably detect asymmetry.

In addition, the grading of recommendations assessment, development, and evaluation (GRADE) was used to assess the quality of evidence for key primary outcomes, with evaluation dimensions including study limitations, inconsistency, indirectness, imprecision, and publication bias. Evidence quality was categorized into four levels: high, moderate, low, and very low. RCTs were initially assigned a high‐quality rating by default, which was adjusted based on the aforementioned downgrading factors. For outcomes with few included studies (≤ 2) or rare events, the quality of evidence was downgraded to at least low due to imprecision.

## 3 Results

### 3.1 Search Results

**Figure 1:**
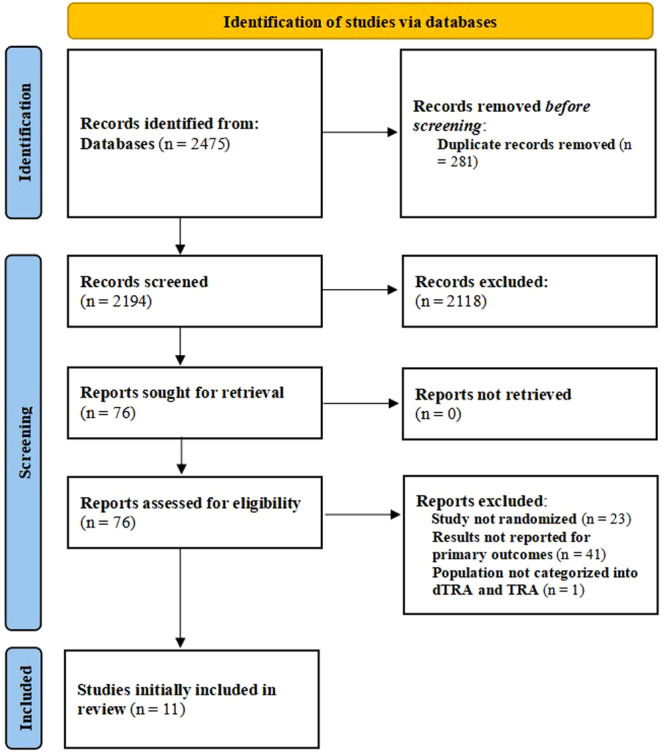
Flow diagram of the search for studies included in the meta analysis according to the Preferred Reporting Items for Systematic Reviews and Meta‐Analyses (PRISMA).

A total of 2475 documents were initially retrieved (1767 from the PubMed database and 708 from the WOS database). After the initial screening of the 2475 citations, 281 duplicates were excluded, leaving 2194 for the next screening step. By browsing the titles and abstracts, 2118 ineligible citations were excluded. For the remaining 76 citations, we performed a detailed assessment and found that 23 citations were not randomized, 41 citations did not report the primary outcome, and 1 Inclusion of study population subgroup that was ineligible. Ultimately, a total of 11 RCTs mentioning hand dysfunction after dTRA and TRA were included in this study [16, 17, 18, 19, 20, 21, 22, 23, 24, 25, 26]. Figure 1 shows the PRISMA (Preferred Reporting Items for Systematic Review and Meta‐Analyses) flow diagram for retrieving and screening studies.

### 3.2 Risk of Bias Assessment

The risk of bias of the included studies was assessed (Supporting Information Figures S1,S2). Among the 11 studies, seven studies had a “high risk of bias” in fewer than two items [16, 17, 18, 19, 20, 25, 26], and one had a “high risk of bias” in four items [21].

### 3.3 Characteristic Information of the Included Studies

Supplementary Table 1 shows the inclusion and exclusion criteria for each of the included studies, all of which were RCTs. Eight of the studies were registered with ClinicalTrials. gov and received a registration number [16, 17, 18, 19, 20, 23, 25, 26], four were multicenter studies [17, 18, 22, 24], and six were clearly stated to be open‐label at the time of study design [16, 17, 18, 24, 25, 26].

Table 1 shows patient characteristics and procedure‐related information. A total of 6903 participants were included, with 1307 participants in the largest study [17] and 64 participants in the smallest study [25]. The mean age of the participants was 62.8 years, and 66.9% of the participants were male. The puncture site of the distal radial artery (DRA) was specified in all studies (Table 2). Six studies used an anatomic snuffbox (AS) as the puncture site [16, 19, 20, 23, 25, 26], one study chose the dorsum of the hand as the puncture site [21], and two studies utilized both of these puncture sites [17, 18]. The remaining studies used the first metacarpal space of the hand as the puncture site [22, 24].

**Table 1:** Demographic characteristics of the included studies.

Study ID	Number (*n*)	Mean age (years)	Male (%)
Al‐Azizi K (2024) [16]	300	66.6	75.3
Aminian A (2022) [17]	1307	68.1	72.5
Babunashvili AM (2024) [18]	850	62.9	65.4
Chen T (2024) [19]	801	66.0	56.2
Daralammouri Y (2022) [20]	209	57.4	74.1
Gupta M (2023) [21]	420	54.5	59.0
Kılıç R (2023) [22]	540	60.4	67.2
Koziński Ł (2023) [23]	400	66.9	60.3
Sharma AK (2020) [24]	970	55.0	59.3
Tehrani BN (2024) [25]	64	67.5	70.3
Tsigkas G (2022) [26]	1042	66.0	75.9

**Table 2:** Procedural characteristics of the included studies.

Study ID	DRA puncture location	Right access side (*n*)	Puncture technique	Access sheath size	Hemostasis system
Al‐Azizi K (2024) [16]	Anatomical snuffbox	293	Modified Seldinger	5/6 French slender radial sheaths	TRA: TR BANDdTRA: Preclude SYNC DISTAL BAND
Aminian A (2022) [17]	Anatomical snuffbox/Dorsum of the hand	1053	Seldinger/Modified Seldinger	6 French Glidesheath Slender (Terumo)	TRA: Air‐filled closure device dTRA: According to hospital practice
Babunashvili AM (2024) [18]	Anatomical snuffbox/Dorsum of the hand	534	Modified Seldinger	Hydrophilic coated introducer sheaths (Terumo, Merit, Lepu)Length not exceeding 16 cm5 French (18.5%)6 French (81.2%)7 French (0.3%)	TRA: Dedicated closure device (Details not available) dTRA: Compression bandage
Chen T (2024) [19]	Anatomical snuffbox	797	Seldinger	6 French (99.5%)7 French (0.5%)	TRA: TR BANDdTRA: Gauze and elastic bandages
Daralammouri Y (2022) [20]	Anatomical snuffbox	NA	NA	6 French	TRA: TR BANDdTRA: Safeguard band
Gupta M (2023) [21]	Dorsum of the hand	NA	Modified Seldinger	NA	TRA: Transparent Radial Artery BANDdTRA: Gauze and elastic bandages
Kılıç R (2023) [22]	The intersection of the thumb and index finger	NA	Seldinger	6 French	TRA: Transradial, BANDdTRA: NA
Koziński Ł (2023) [23]	Anatomical snuffbox	240	Modified Seldinger	5 French/6 French Glidesheath Slender (47.3%)6 French/7 French Glidesheath Slender (52.7%)	TRA: Gauze and elastic bandages dTRA: Gauze and elastic bandages
Sharma AK (2020) [24]	The intersection of the thumb and index finger	NA	Seldinger	5 French	TRA: TR BANDdTRA: Gauze and elastic bandages
Tehrani BN (2024) [25]	Anatomical snuffbox	46	Seldinger/Modified Seldinger	6/7 French Glidesheath Slender (Terumo)	TRA: TR BAND (Terumo) dTRA: Safeguard Compression Device (Merit Medical, Jordan, UT)
Tsigkas G (2022) [26]	Anatomical snuffbox	1042	Seldinger	Standard 11 cm long radial artery sheaths 5 French (62.5%)6 French (36.9%)7 French (0.6%)	TRA: TR BANDdTRA: TR BAND (When the TR Band didn't fit the snuffbox area well, a plastic strip was used)

### 3.4 Outcomes

An analysis of 11 RCTs revealed significant heterogeneity in the measurement of hand dysfunction and the follow‐up time. The heterogeneity in measurement heterogeneity is reflected by the fact that Al‐Azizi K and Babunashvili et al. used different hand function kits [16, 18]. Other studies mentioned the use of questionnaires (including DASH, Quick DASH, VAS, Borg, and NRS scores) to specify hand motor and sensory function [17, 20, 22, 25, 26]. The heterogeneity in the follow‐up time is reflected in the fact that some studies assessed the follow‐up time only within 24 h after the procedure. Koziński et al. performed follow‐up at 2 months after the procedure [23], whereas Babunashvili et al. performed follow‐up at 1 week, 1 month, 6 months, and 12 months [18]. All the results are summarized in Tables 3 and 4. Notably, although monofilament testing was specified in our protocol, none of the included studies utilized this assessment method.

### 3.5 Grip Strength, Pinch Strength and Questionnaire for Measuring Hand Function

Three studies reported hand function measurements by instruments and questionnaires after TRA and dTRA (Table 3 **and** Supplementary Information Table S2). The studies by Babunashvili and Al‐Azizi et al. both provided data on grip and pinch strength at baseline and at the 12‐month follow‐up [16, 18], whereas Tehrani et al. reported data on DASH scores only at the follow‐up at 3 months after the procedure [25]. In the study by Babunashvili et al., grip and pinch strength in both the TRA and dTRA groups improved at the December follow‐up compared with baseline. Al‐Azizi et al. came to similar conclusions, but their study revealed that Quick DASH scores at the 12‐month follow‐up showed a mild degree of hand dysfunction and even a slight improvement from baseline.

**Table 3:** Comparison of hand function and complications between TRA and dTRA.

Study ID	Measure of hand function and vascular complication	Baseline and follow‐up	Result
Al‐Azizi K (2024) [16]	Hand function: Grip test, pinch test, and Quick DASH questionnaire. Vascular complication: Doppler ultrasound	Baseline	TRAGrip Test: 24.0 (17.0–31.3); Pinch Test: 7.1 (5.4–8.2); Quick DASH: 4.6 (0.0–15.9); dTRA Grip Test: 26.0 (18.9–36.0); Pinch Test: 7.9 (5.7–9.1); Quick DASH: 4.6 (0.0–13.6)
		24 h after the procedure	TRABleeding: 2/150 (1.4%); Hematoma: 1/150 (0.7%); dTRABleeding: 0/150 (0.0%); Hematoma: 0/150 (0.0%)
		1 year after the procedure	TRAdRAO: 2/104 (1.9%); RAO: 1/104 (1.0%); Grip Test: 25.3 (18.8–31.7); Pinch Test: 6.9 (5.0–8.0); Quick DASH: 4.6 (0.0–12.0); dTRAdRAO: 0/112 (0.0%); RAO: 0/112 (0.0%); Grip Test: 29.2 (18.0–37.2); Pinch Test: 8.0 (6.0–9.0); Quick DASH: 2.7 (0.0–13.6)
Aminian A (2022) [17]	Hand function: VAS questionnaire. Vascular complication: Doppler ultrasound	48 h after the procedure	TRARAO: 6/657 (0.9%); dRAO: NABleeding: 36/657 (5.5%); VAS: 2.0 (0.0–3.0); Radial spasm: 18/657 (2.7%); Bleeding: 36/657 (5.5%); dTRARAO: 2/650 (0.3%); dRAO: 3/650 (0.5%); Bleeding: 44/650 (6.8%); VAS: 2.0 (0.0–4.0); Radial spasm: 35/650 (2.7%); Bleeding: 44/650 (6.8%)
Babunashvili AM (2024) [18]	Hand function: Grip test, pinch test, and VAS questionnaire. Vascular complication: Doppler ultrasound	Baseline	TRAGrip Test: 40.0 (30.0‐49.0)Pinch Test: 10.0 (7.0‐12.0)dTRAGrip Test: 38.0 (28.0‐48.0)Pinch Test: 9.5 (7.0‐12.5)
		24 h after the procedure	TRARAO: 15/418 (3.6%); Bleeding: 86/418 (20.5%); Hematoma: 113/418 (27.0%); VAS: 0.0 (0.0–2.0); Pseudoaneurysm: 1/418 (0.2%); Arteriovenous fistula: 0/418 (0.0%); Radial spasm: 100/418 (23.9%); dTRARAO: 5/432 (1.2%); Bleeding: 14/432 (3.2%); Hematoma: 39/432 (9.0%); VAS: 1.0 (0.0–2.0); Pseudoaneurysm: 0/393 (0.0%); Arteriovenous fistula: 0/432 (0.0%); Radial spasm: 102/432 (23.6%)
		1 year after the procedure	TRARAO: 28/418 (6.7%); Grip Test: 42.0 (30.0–51.0); Pinch Test: 11.0 (8.5–14.0); dTRARAO: 11/418 (2.5%); Grip Test: 40.0 (28.0–50.0); Pinch Test: 10.0 (30.0–49.0)
Chen T (2024) [19]	Hand function: VAS questionnaire. Vascular complication: Doppler ultrasound	24 h after the procedure	TRARAO: 27/403 (6.7%); Bleeding: 24/403 (3.0%); Hematoma: 18/403 (4.5%); VAS: 0.0 (0.0–1.0); Hand numbness: 37/403 (9.2%); Pseudoaneurysm: 1/403 (0.2%); Arteriovenous fistula: 0/403 (0.0%); dTRARAO: 10/398 (2.5%); Bleeding: 6/398 (1.5%); Hematoma: 18/398 (4.5%); VAS: 0.0 (0.0–1.0); Hand numbness: 81/398 (20.4%); Pseudoaneurysm: 0/398 (0.0%); Arteriovenous fistula: 0/398 (0.0%)
		3 months after the procedure	TRARAO: 12/403 (3.3%); dTRARAO: 3/398 (0.8%)
Daralammouri Y (2022) [20]	Hand function: NRS questionnaire. Vascular complication: Doppler ultrasound	24 h after the procedure	TRARAO: 2/105 (1.9%); Hematoma: 6/105 (5.8%); Dissection: 0/105 (0.0%); Radial spasm: 3/105 (2.9%); Bleeding: 0/105 (0.0%); NRS: 1.6 ± 1.6; dTRARAO: 0/104 (0.0%); Hematoma: 3/104 (2.8%); Dissection: 0/104 (0.0%); Radial spasm: 4/104 (3.8%); Bleeding: 0/104 (0.0%); NRS: 2.1 ± 2.3
Gupta M (2023) [21]	Observation and physical examination	24 h after the procedure	TRARAO: 27/210 (12.9%); Hematoma: 17/210 (5.8%); Radial spasm: 2/210 (1.0%); Persistent pain: 29/210 (13.8%); Hand clumsiness: 19/210 (9.0%); dTRARAO: 4/210 (1.9%); Hematoma: 21/210 (10.0%); Radial spasm: 3/210 (1.4%); Persistent pain: 2/210 (1.0%); Hand clumsiness: 1/210 (0.5%)
Kılıç R (2023) [22]	Hand function: VAS questionnaire. Vascular complication: Observation and physical examination	After the procedure (unclear)	TRAVAS: 3.9 ± 1.9; dTRAVAS: 4.9 ± 2.1
Koziński Ł (2023) [23]	Hand function: Observation, physical examination, and ultrasound. Vascular complication: Doppler ultrasound	24 h after the procedure	TRARAO: 9/200 (4.5%); Hematoma: 49/200 (24.5%); Bleeding: 1/200 (0.5%); Pseudoaneurysm: 1/200 (0.5%); Dissection: 3/200 (1.5%); Radial spasm: 9/200 (4.5%); Local neuropathy: 58/200 (29.0%); Thumb numbness: 58/200 (29.0%); Arteriovenous fistula: 0/200 (0.0%); dTRARAO: 5/200 (2.5%); Hematoma: 40/200 (20.0%); Bleeding: 0/200 (0.0%); Pseudoaneurysm: 0/200 (0.0%); Dissection: 19/200 (9.5%); Radial spasm: 38/200 (19.0%); Local neuropathy: 29/200 (14.5%); Thumb numbness: 29/200 (14.5%); Arteriovenous fistula: 4/200 (0.2%)
		2 months after the procedure	TRARAO: 6/197 (3.0%); Local neuropathy: 1/197 (0.5%); dTRARAO: 5/199 (2.5%); Local neuropathy: 0/199 (0.0%)
Sharma AK (2020) [24]	Hand function: Observation and physical examination. Vascular complication: Observation, physical examination, and Doppler ultrasound	After the procedure (unclear)	TRARAO: 63/485 (13.0%); Hematoma: 38/485 (7.8%); Radial spasm: 58/485 (12.0%); Persistent pain: 68/485 (14.0%); Hand clumsiness: 44/485 (9.1%); dTRARAO: 10/485 (2.1%); Hematoma: 48/485 (9.9%); Radial spasm: 5/485 (1.0%); Persistent pain: 5/485 (1.0%); Hand clumsiness: 2/485 (0.4%)
Tehrani BN (2024) [25]	Hand function: DASH and Borg questionnaire. Vascular complication: Observation, physical examination, and Doppler ultrasound	3 months after the procedure	TRARAO: 0/27 (0.0%); Pseudoaneurysm: 0/27 (0.0%); Dissection: 0/27 (0.0%); Hematoma: 0/31 (0.0%); Borg: 10.6 ± 2.6; DASH: 8.0 ± 12.9; dTRARAO: 0/31 (0.0%); Pseudoaneurysm: 0/31 (0.0%); Dissection: 0/31 (0.0%); Hematoma: 0/33 (0.0%); Borg: 11.5 ± 3.4; DASH: 5.7 ± 10.2
Tsigkas G (2022) [26]	Hand function: VAS questionnaire. Vascular complication: Observation, physical examination, and Doppler ultrasound	2 months after the procedure	TRARAO: 31/524 (7.9%); Pseudoaneurysm: 16/524 (4.1%); Arteriovenous fistula: 0/524 (0.0%); Bleeding: 0/524 (0.0%); Hematoma: 8/524 (1.5%); Radial spasm: 0/524 (0.0%); VAS: 3.0 (2.0–5.0); dTRARAO: 15/518 (3.7%); Pseudoaneurysm: 7/518 (1.7%); Arteriovenous fistula: 2/518 (0.5%); Bleeding: 0/518 (0.0%); Hematoma: 4/518 (0.8%); Radial spasm: 1/518 (0.2%); VAS: 3.0 (2.0–5.0)

### 3.6 Hand Sensory Dysfunction

**Figure 2:**
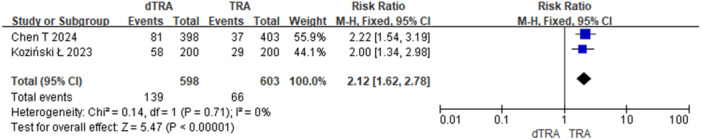
Main analysis of hand numbness in included studies comparing dTRA and TRA (Mantel–Haenszel fixed‐effect).

**Figure 3:**
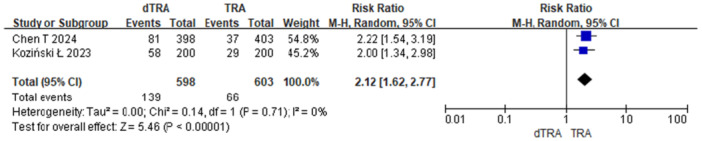
Sensitivity analysis of hand numbness in included studies comparing dTRA and TRA (Mantel–Haenszel random‐effects).

**Figure 4:**
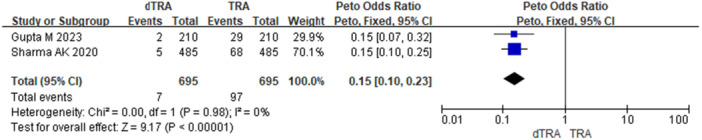
Main analysis of hand persistent pain in included studies comparing dTRA and TRA (Peto fixed‐effect).

**Figure 5:**
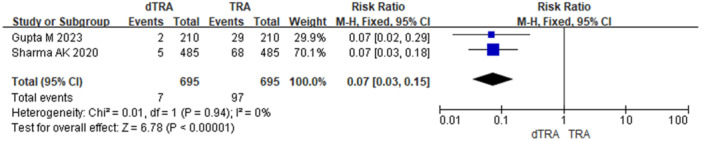
Sensitivity analysis of hand persistent pain in included studies comparing dTRA and TRA (Mantel–Haenszel fixed‐effect).

**Figure 6:**
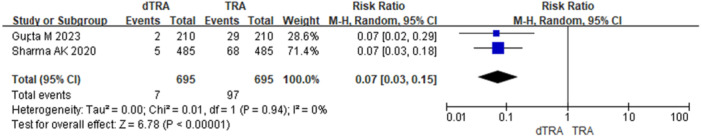
Sensitivity analysis of hand persistent pain in included studies comparing dTRA and TRA (Mantel–Haenszel random‐effects).

Postoperative hand sensory dysfunction includes numbness and pain. A total of 2 studies reported postoperative hand numbness after dTRA versus TRA (23.2% *vs*. 10.9%, RR = 2.12, 95% CI: 1.62–2.77, *p* < 0.001, NNTH = 8) (Figure 2). Sensitivity analysis using a random‐effects model yielded consistent results (Figure 3). A total of 10 studies assessed postoperative patient pain. Only two studies compared persistent postoperative pain between dTRA and TRA (1.0% *vs*. 14.0%). Given the low event rates, the Peto method was employed as the primary analysis, which demonstrated a significant reduction with dTRA (RR = 0.15, 95% CI: 0.10–0.23, *p* < 0.001, NNTB = 8) (Figure 4). Sensitivity analyses using Mantel–Haenszel fixed‐effect and random‐effects models yielded consistent results (Figures 5 and 6).

Postoperative pain was assessed using different scales with varying ranges. The Visual Analog Scale (VAS, range 0–10), Numeric Rating Scale (NRS, range 0–10), and Borg Scale (range 0–20). These scales differ in both range and construct validity: VAS and NRS are specifically designed for pain intensity measurement, while the Borg Scale was originally developed for rating perceived exertion and dyspnea. When standardized to the percentage of scale range, the magnitude of pain differences was comparable across instruments. Specifically, Daralammouri et al. assessed pain symptoms at 24 h after the procedure by the NRS [(2.1 ± 2.3) *vs.* (1.6 ± 1.6)], representing a difference of 0.5 points (5.0% of the 0–10 scale); Tehrani et al. assessed pain symptoms at 3 months postoperatively by Borg scale [(11.5 ± 3.4) *vs.* (10.6 ± 2.6)], representing a difference of 0.9 points (4.5% of the 0–20 scale); and the remaining studies assessed pain symptoms by the VAS scale, with differences typically < 1.0 point (< 10% of the scale range) (Table 3).

### 3.7 Hand Motor Dysfunction

**Figure 7:**
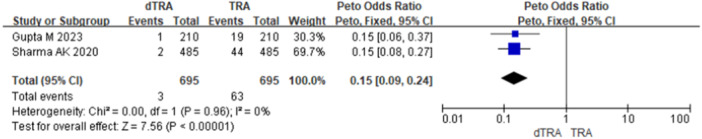
Main analysis of hand clumsiness in the included studies comparing dTRA and TRA (Peto fixed‐effect).

**Figure 8:**
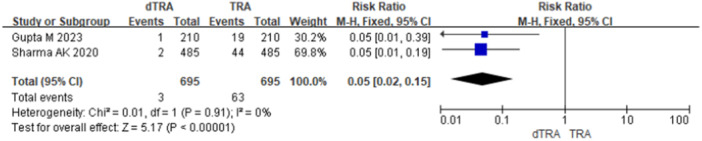
Sensitivity analysis of hand clumsiness in included studies comparing dTRA and TRA (Mantel–Haenszel fixed‐effect).

**Figure 9:**
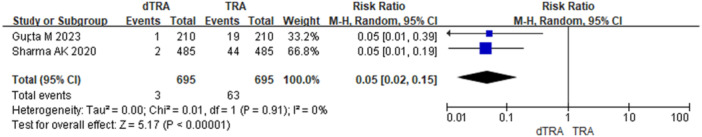
Sensitivity analysis of hand clumsiness in included studies comparing dTRA and TRA (Mantel–Haenszel random‐effects).

Based on limited evidence, two studies reported the occurrence of hand clumsiness after TRA and dTRA (0.4% *vs*. 9.1%). Given the low event rates, the Peto method was employed as the primary analysis, which demonstrated a significant reduction with dTRA (RR = 0.15, 95% CI: 0.09–0.24, *p* < 0.001, NNTB = 11) (Figure 7). Sensitivity analyses using Mantel–Haenszel fixed‐effect and random‐effects models yielded consistent results (Figures 8 and 9). Gupta et al. reported that at 24 h after the procedure, only 1 patient (1/210, 0.5%) who underwent dTRA developed hand clumsiness. Similarly, Sharma et al. reported that two patients (2/485, 0.4%) who underwent dTRA had hand clumsiness, but the follow‐up time was not mentioned (Table 3).

### 3.8 Nerve Injury

One of the included studies reported localized neuropathy after TRA or dTRA [23]. Koziński et al. reported incidences of 29.0% and 14.5%, respectively, within 24 h after the procedure. The incidence rates at 2 months postoperative follow‐up were 0.5% and 0.0%, respectively.

### 3.9 Other Outcomes

Table 4 summarizes the data on complications within 24 h after TRA and dTRA. Ten studies reported RAO incidence. After excluding one study with zero events in both arms, meta‐analysis of nine studies showed dTRA significantly reduced RAO risk compared with TRA (1.6% *vs*. 5.8%, RR: 0.29, 95% CI: 0.21–0.39, *p* < 0.001) (Supporting Information Figures S3,S4). We also perform a meta‐analysis of other vascular complications (Supporting Information Figures 5–7). Table 5 compares TRA and dTRA in terms of other procedure characteristics.

**Table 4:** Summary of the pooled results within 24 h after TRA and dTRA.

Hand dysfunction and vascular complication	Number of study	TRA			dTRA		
		Events	Total	Percent	Events	Total	Percent
Hand clumsiness	2	63	695	9.1%	3	695	0.4%
Hand numbness	2	66	603	10.9%	139	598	23.2%
Local neuropathy	1	58	200	29.0%	29	200	14.5%
Persistent pain	2	97	695	14.0%	7	695	1.0%
Radial artery occlusion	9	181	3106	5.8%	51	3109	1.6%
Hematoma	8	250	2495	10.0%	173	2497	6.9%
Arteriovenous fistula	2	0	724	0.0%	6	718	0.8%
Pseudoaneurysm	4	19	1545	1.3%	7	1509	0.5%
Dissection	3	3	332	0.9%	19	335	0.6%
Radial spasm	7	190	2599	7.3%	188	2599	7.2%
Bleeding	7	149	2457	6.1%	64	2452	2.6%

**Table 5:** Comparison of other outcomes between TRA and dTRA.

Study ID	Radial artery accessed		Single puncture		Puncture time (s)		Procedure time (min)		Contrast volume (ml)		Hemostasis time (min)	
	TRA	dTRA	TRA	dTRA	TRA	dTRA	TRA	dTRA	TRA	dTRA	TRA	dTRA
Al‐Azizi K (2024) [16]	144/150	145/150	NA	NA	NA	NA	NA	NA	NA	NA	NA	NA
Aminian A (2022) [17]	NA	NA	NA	NA	NA	NA	24.0 (14.0–42.0)	27.0 (15.0–45.0)	95.2 ± 73.1	91.8 ± 65.0	180.0 (134.0–292.0)	153.0 (105.0–242.0)
Babunashvili AM (2024) [18]	414/418	412/432	NA	NA	35.0 (23.0–56.0)	44.0 (28.0–78.5)	20.0 (8.0–35.0)	20.0 (10.0–35.0)	NA	NA	180.0 (120.0–460.0)	156.5 (125.0–195.0)
Chen T (2024) [19]	397/403	382/398	338/403	282/398	60.0 (50.0–60.0)	60.0 (50.0–90.0)	30.0 (15.0–47.0)	25.0 (15.0–38.5)	50.0 (50.0–110.0)	50.0 (50.0–90.0)	NA	NA
Daralammouri Y (2022) [20]	103/105	102/104	NA	NA	20.0 ± 18.4	56.3 ± 58.3	18.2 ± 15.5	13.1 ± 11.2	72.1 ± 47.7	58.0 ± 36.4	134.3 ± 50.1	114.8 ± 44.4
Gupta M (2023) [21]	206/210	202/210	193/210	164/210	96.0 ± 48.0	72.0 ± 36.0	20.0 ± 3.3	21.0 ± 2.9	19.0 ± 5.0	21.0 ± 4.0	24.0 ± 6.2	28.0 ± 7.9
Kılıç R (2023) [22]	NA	NA	NA	NA	41.8 ± 12.6	53.0 ± 16.4	41.4 ± 12.0	40.7 ± 12.4	NA	NA	NA	NA
Koziński Ł (2023) [23]	198/200	199/200	170/200	161/200	80.0 (58.0–127.0)	140.0 (85.0–322.0)	16.5 (9.7–31.5)	18.9 (11.0–34.1)	47.0 (30.0–97.0)	40.0 (25.0–87.0)	NA	NA
Sharma AK (2020) [24]	475/485	466/485	446/485	378/485	NA	NA	NA	NA	NA	NA	24.0	28.0
Tehrani BN (2024) [25]	NA	NA	NA	NA	55.5 ± 40.4	42.4 ± 13.2	NA	NA	93.0 ± 64.1	79.2 ± 48.0	160.9 ± 86.2	157.4 ± 77.3
Tsigkas G (2022) [26]	497/524	408/518	NA	NA	75 (50–120)	120 (60–251)	11.0 (8.0–19.0)	14.0 (9.0–23.0)	70.0 (53.0–104.0)	71.0 (50.0–118.0)	NA	NA

### 3.10 Evaluation of Evidence Quality

This study used the GRADE to assess the quality of evidence for the primary outcome.

The quality of evidence for hand numbness was moderate. Although the pooled results indicate that dTRA significantly increases the risk of numbness, this outcome is based on only two studies, with a relatively limited sample size and methodological heterogeneity (different assessment time points and tools used across studies). Therefore, the evidence was downgraded to moderate quality.

The quality of evidence regarding hand clumsiness and persistent pain was low. Only two studies were included for each of these outcomes, and the incidence rates were extremely low. Although the effect sizes were significant and heterogeneity was low, there was substantial imprecision (small sample sizes, narrow confidence intervals based on rare events) and potential publication bias (small‐sample studies are more likely to report positive results). Therefore, the quality of evidence was downgraded to low. Precise effect sizes should be interpreted with caution, but the directional conclusion (that dTRA reduces risk) is relatively reliable.

The quality of evidence regarding nerve damage was very low. This finding is based on only one study; given the small sample size and lack of replication, random error cannot be ruled out. Therefore, the evidence is rated as very low quality and should be used for reference only.

## 4 Discussion

### 4.1 Main Findings

To our knowledge, this study is the first to compare and systematically present the incidence and clinical manifestations of hand dysfunction after dTRA and TRA interventions, as well as to summarize the incidence of postoperative vascular complications. However, due to significant heterogeneity among the included studies in terms of definitions of hand function dysfunction, assessment methods, and follow‐up duration, the following findings should be regarded as exploratory results. Based on limited evidence, this study preliminarily suggests that hand sensory impairment may be a relatively common form of hand dysfunction. A pooled analysis of only two studies showed that, compared with TRA, dTRA was associated with a potentially higher risk of hand numbness at 24 h post‐operation (23.2% *vs*. 10.9%, RR = 2.21, 95% CI: 1.62–2.77, *p* < 0.001, NNTH = 8), whereas the risk of persistent pain at 24 h may be lower (1.0% *vs*. 14.0%, RR = 0.15, 95% CI: 0.10–0.23, *p* < 0.001, NNTB = 8).

When evaluating hand dysfunction, in addition to assessing hand function itself, other factors should also be taken into consideration, including vascular diameter, vascular endothelial function, the presence of peripheral vascular disease, diabetes, the number of punctures, sheath size, and the hemostasis system.

It is worth noting that not all outcomes carry equal clinical weight. Persistent pain and hand clumsiness—which directly impair patients’ ability to perform daily activities such as grasping objects, writing, or manipulating tools—may be more clinically meaningful than transient sensory symptoms (such as temporary numbness within 24 h). The former reflects functional disability that can significantly affect quality of life and occupational performance, whereas the latter often represents temporary, self‐limiting symptoms related to local compression or hematoma that resolve without intervention. However, current evidence is insufficient to definitively establish this hierarchy, as the studies reporting these outcomes employed different examination techniques and heterogeneous follow‐up protocols.

### 4.2 Mechanisms of Hand Dysfunction

The results of this study revealed that most of the study puncture points were selected in the AS during the dTRA operation. The AS is located at the base of the thumb, which is a triangular area surrounded by the tendons of the extensor pollicis brevis (EPB), the abductor pollicis longus (APL), and the extensor pollicis longus (EPL). The triangular area contains the DRA, cephalic vein (CV), and superficial branch of the radial nerve (SBRN). Notably, this anatomical arrangement may explain the seemingly contradictory findings regarding sensory and motor outcomes: specifically, why dTRA increased hand numbness (23.2% vs. 10.9%) while reducing hand clumsiness (0.4% vs. 9.1%). The close proximity of the SBRN to the dTRA puncture site predisposes to direct mechanical irritation or transient compression during catheterization, accounting for the higher incidence of hand sensory dysfunction. In contrast, TRA is the radial artery distal to the SBRN bifurcation, sparing this sensory branch. Conversely, the more superficial location of the DRA in the AS—surrounded by bony structures—enables shorter compression times and avoids venous congestion during hemostasis, thereby significantly reducing the risk of hematoma formation and RAO [9]. Since hand motor dysfunction is strongly associated with RAO and compartment compression from hematomas, dTRA effectively reduces vascular‐mediated motor impairment while incurring a higher risk of nerve‐mediated sensory symptoms. Furthermore, the temporal dissociation between these outcomes should be considered: the increased numbness with dTRA primarily occurs within 24 h post‐procedure, likely representing acute neuropraxia or local anesthetic effect that is typically self‐limiting [23], whereas clumsiness reflects persistent functional impairment from chronic vascular complications. Thus, dTRA trades a transient, self‐limiting sensory symptom for a lower risk of chronic motor disability.

During puncture and catheterization, adjacent tendons or nerves may be directly damaged, resulting in motor and sensory dysfunction [27]. In a real‐world study, researchers evaluated patients who underwent left dTRA and reported that neuropathy was detected in two patients (2/141) during ultrasound follow‐up 1 month after the procedure [12]. In contrast, Zankl et al. reported only one case of radial nerve injury in a study of 488 participants who underwent TRA [28]. A previous systematic review of upper limb dysfunction after TRA reported that the overall incidence of nerve damage after TRA surgery was 0.16% [13]. In this study, we reviewed previous RCTs and found that only one study reported the incidence rates of neuropathy at 24 h after dTRA and TRA, which were 14.5% and 29.0%, respectively [23]. This result is significantly greater than the data from the other studies mentioned above, and this difference may be related to differences in follow‐up time. Notably, this study conducted a follow‐up at 2 months after the procedure, and the results revealed that the incidence of neuropathy in the dTRA group and TRA group decreased to 0.0% and 0.5%, respectively. The exact mechanism of hand dysfunction (motor or sensory dysfunction) after dTRA and/or TRA intervention is currently unclear and warrants further discussion.

Local hematoma, tissue edema, or prolonged postoperative pressure may affect hand function [29]. During TRA or dTRA interventional procedures, direct vascular injury is the primary cause of hematoma formation. In particular, anatomical variability or improper puncture technique may increase the risk of local bleeding and result in compression of the surrounding arteries and nerves [30]. Arterial compression can easily lead to local ischemia in surrounding tissues. Under ischemic conditions, muscle cells release large amounts of lactic acid, which may stimulate sensory neurons and cause ischemic pain [31]. Nerve compression can directly cause hand sensory dysfunction in the hands, resulting in symptoms such as numbness and pain [32]. For the assessment of hematoma, previous studies have used the modified early discharge after transradial stenting of coronary arteries study (mEASY) method [26]. Notably, in their study, there was no statistically significant difference between the TRA group and the dTRA group in terms of mEASY ≥ II hematoma, but the dTRA group had a significantly shorter compression hemostasis time. This may be attributed to the anatomical characteristics of the DRA. The DRA is located in the AS and is surrounded by numerous bony structures with relatively superficial blood vessels, which makes postoperative compression hemostasis easier [33]. Additionally, unlike TRA, dTRA does not block veins during postoperative compression hemostasis, thereby significantly reducing the risk of hand congestion [34].

RAO is a common complication of TRA intervention procedures and may be associated with hand dysfunction [35]. This may be related to the prolonged compression time after TRA. Compared with patients who underwent dTRA, those who underwent TRA had a greater risk of developing hand numbness [36]. The results of this study led to a similar conclusion. Amoroso et al. suggested that RAO is associated with persistent upper limb dysfunction, and a study reported that thinner catheters appear to reduce the incidence of upper limb dysfunction in the short term [37]. Rashid et al. also found through meta‐analysis that the outer diameter of the vascular sheath is positively correlated with the incidence of RAO [7].

However, previous studies have reached the opposite conclusion, finding that chronic RAO after TRA is not associated with decreased hand grip strength or thumb and index finger pinch strength [38]. Van Leeuwen et al. reached a similar conclusion [39]. However, we cannot ignore the potential risks of this common complication. In certain special cases, RAO may lead to severe ischemia of the hand or even amputation [40]. Notably, the anatomical structure of the DRA is unique. After the dTRA procedure, the hemostasis time is shorter than that of the TRA procedure, which significantly reduces patient discomfort and vascular complications caused by prolonged compression, especially RAO [9, 35]. In addition, AVFs may also affect hand function. In a previous case, a patient developed an AVF after undergoing TRA, which led to hand dysfunction, specifically manifested as a decrease in two‐point discrimination, monofilament testing, and pinch strength [41].

Postoperative pain is a common reaction to tissue damage. Although transradial intervention is a minimally invasive procedure, postoperative pain may still occur [22]. Studies by Zwaan et al. have shown that the incidence of upper limb dysfunction after PCI via TRA is 9.6%, with severe acute pain being an important symptom [42]. Previous studies have reported that the incidence of severe pain after PCI following TRA is 4‐10%, which has a significant negative impact on patients’ daily life and work capacity [43]. The occurrence of acute pain at the puncture site may be related to the patient's preoperative anxiety, swelling of the hand after hemostasis, and postoperative complications at the puncture site (bleeding or hematoma) [43]. Compared with TRA, the smaller internal diameter of the artery leads to an increase in the number of punctures, resulting in greater postoperative pain after dTRA [22]. However, this study revealed that although dTRA patients experienced a greater degree of postoperative pain, they had a lower risk of developing persistent pain.

### 4.3 Hand Function Assessment Methods

Currently, there is no consensus on the best method for assessing hand function after dTRA and TRA interventions. Existing studies have used a variety of testing methods. In terms of objective evaluation, researchers have used ultrasound and electromyography to assess nerve injury [44] and hand function kits to measure grip strength, pinch strength, two‐point discrimination ability, and monofilament [18, 45]. In terms of subjective evaluation, some studies have used questionnaires to assess postoperative pain and upper limb function [16, 18]. Recent studies have also used hand thermal imaging to assess hand function after TRA intervention [46]. However, different assessment methods have their own advantages and limitations.

Ultrasound and electromyography are important tools for assessing nerve damage, providing relatively objective evidence of nerve damage [47]. Ultrasound examination is a non‐invasive imaging method that can visually display the morphology and structure of nerves and assess nerve continuity and the location of damage. High‐frequency ultrasound is particularly suitable for examining superficial nerves such as the radial nerve and median nerve. It can clearly show the course of the nerve and whether there are any abnormal echoes, thereby determining whether the nerve is damaged [48]. The advantages of ultrasound examination include ease of operation, non‐invasiveness, low cost, and the ability to dynamically observe changes in nerves. In the ANTARES study, 200 patients who underwent dTRA were examined by ultrasound within 24 h after the procedure, and the results revealed that 58 patients experienced local nerve damage [23]. Notably, however, ultrasound can display only morphological information about nerves and cannot reflect nerve conduction function. Electromyography, as a complementary tool to ultrasound, effectively addresses this limitation [47]. However, electromyography is complicated to perform and requires specialized personnel and equipment, which limits its widespread clinical application.

Hand function kits, including a grip strength meter, pinch strength meter, and monofilaments, can be used to quantitatively or semi‐quantitatively assess hand sensation and motor function after dTRA or TRA intervention [36]. Hand function testing equipment is simple and easy to operate, capable of comprehensively assessing hand strength and sensation, and highly repeatable when operated under standardized measurement postures [49]. However, because of the influence of multiple factors on the hand function, it cannot be accurately evaluated using a single variable. A multicenter study by Sgueglia et al. assessed hand function in patients with dTRA before and after the procedure using multiple methods (including grip strength, sensory monofilaments, and questionnaire scales). The results revealed that dTRA did not impair hand function, and some indicators even showed a slight improvement trend during postoperative follow‐up [36]. Similar conclusions were reached in studies by Babunashvili et al. In both the TRA group and the dTRA group, patients’ grip strength and pinch strength decreased at the end of the procedure but improved over time [18]. According to the results of this study, none of the included RCTs used monofilament assessments of hand sensation, indicating that further research is needed to clarify postoperative sensory function after dTRA and TRA. Figure 6.

It is important to acknowledge that pain outcomes were assessed using instruments with different measurement properties. The Borg Scale (range 0–20), originally designed for rating perceived exertion and dyspnea, was applied to postoperative discomfort in one study [25], while others used pain‐specific scales (VAS/NRS, range 0–10). This creates two sources of variation: (1) scale range differences (a 1‐point change on NRS represents 10% of the scale, while 1 point on Borg represents only 5%), and (2) construct differences (Borg may capture fatigue/discomfort rather than sharp pain). However, when standardized to the percentage of scale range, the direction and magnitude of pain differences were consistent: dTRA showed slightly higher pain scores (4.5–10% of maximum) across all three scale types. This convergence across heterogeneous instruments strengthens the reliability of the finding that dTRA‐associated pain, while statistically detectable, represents a minor increase that is unlikely to be clinically consequential.

In contrast, many questionnaires, such as the DASH, Quick DASH, Levine‐Katz, and CISS questionnaires, are used in clinical practice to assess postoperative upper limb function. These can be completed by patients to provide a comprehensive understanding of hand function. However, questionnaire scales are highly susceptible to subjective influences and have a limited ability to distinguish between the presence or absence of upper limb dysfunction after surgery [50].

The 11 randomized controlled trials included in this study exhibited substantial heterogeneity in the assessment of hand dysfunction, manifested in three main aspects. First, definition heterogeneity: some studies defined hand dysfunction as subjective symptoms (such as pain, numbness), while others adopted objective functional indicators (such as grip strength, pinch strength decline) or imaging examinations (ultrasound, electromyography), lacking unified diagnostic criteria. Second, assessment tool heterogeneity: subjective assessments utilized various questionnaires such as DASH scale, VAS score, and Borg score, whereas objective assessments involved different devices including dynamometers, pinch meters, monofilament tests, two‐point discrimination tests, and ultrasound/electromyography, with variations in sensitivity, specificity, and measurement precision. Third, follow‐up duration heterogeneity: assessment timepoints ranged from 24 h to 12 months postoperatively, where short‐term evaluations may capture acute symptoms (such as puncture‐related pain, numbness due to hematoma compression), while long‐term follow‐up reflects chronic complications (such as RAO‐related ischemia, nerve injury sequelae).

The mentioned heterogeneity limits the reliability of meta‐analysis results, as pooled effect sizes may obscure true differences between assessment strategies, thereby restricting clinical generalizability. For example, hand numbness within 24 h postoperatively is often related to local compression or hematoma and is typically transient, whereas persistent numbness at several months postoperatively may indicate nerve injury—these carry distinctly different clinical implications. Therefore, although current evidence suggests potential differences in hand dysfunction between dTRA and TRA, the optimal assessment strategy cannot be determined due to heterogeneity in assessment methods.

### 4.4 Study Limitations

Although we summarized the current RCTs and pooled the main effect sizes, our study also has several limitations. There is considerable heterogeneity in the study designs and measurement methods, such as the varying methods used to assess hand function, and limited detailed data are available on the assessment of postoperative hand dysfunction. Additionally, the key findings of this study, including hand clumsiness, numbness, and persistent pain, were based solely on pooled analyses of two randomized controlled trials. Although the total sample size for these outcomes was relatively large, the limited number of included studies, coupled with heterogeneity in outcome definitions, examination techniques, and follow‐up durations, suggests that these findings should be interpreted with caution. Furthermore, due to heterogeneity in outcome definitions and follow‐up durations, the NNTB and NNTH values (11 for hand clumsiness, 8 for persistent pain, and 8 for hand numbness) should be interpreted with considerable caution.

## 5 Conclusion

This study conducted a meta‐analysis and narrative summary of all available RCTs through a literature review, comparing dTRA with TRA in terms of hand dysfunction and vascular complications after coronary intervention. Limited evidence suggests that, compared with TRA, dTRA may be associated with lower risks of postoperative hand clumsiness, persistent pain, radial artery occlusion (RAO), and arteriovenous fistula (AVF), but potentially with a higher incidence of postoperative hand numbness. Due to significant heterogeneity in study designs and methodologies, and because key outcomes were based on only a small number of trials. In the future, more high‐quality standardized randomized controlled trials are needed to validate these preliminary findings.

## Author Contributions

Gaojun Cai conceived and designed the study. Feng Li, Ganwei Shi, Chenlei Gu, and Ziyang Chen analyzed and interpreted the data. Tong Zhou conducted the re‐analysis of primary outcomes following reviewer comments and substantially revised the manuscript. Xinyu Fan carried out the data collection and drafted the manuscript. All the authors read and approved the final manuscript.

## Conflicts of Interest

The authors declare no conflicts of interest.

## Supporting information


**Figure S1:** Risk of bias graph.**Figure S2:** Risk of bias summary.**Figure S3:** Pool analysis of radial artery occlusion (RAO) in included studies comparing dTRA and TRA.**Figure S4:** Funnel plot for the endpoint of radial artery occlusion (RAO).**Figure S5:** Pool analysis of hematoma in included studies comparing dTRA and TRA.**Figure S6:** Pool analysis of arteriovenous fistula (AVF) in included studies comparing dTRA and TRA.**Figure S7:** Pool analysis of pseudoaneurysm in included studies comparing dTRA and TRA.

**Table S1:** Design of included studies.**Table S2:** Measures of hand function by grip strength, pinch strength and questionnaires.

## Data Availability

All data relevant to the study are included in the article or uploaded as supplementary information. This is a systematic review and meta‐analysis, and no new primary data were generated.
