# Supplementary material for: Hand Dysfunction After Intervention via Distal Versus Conventional Transradial Access: A Meta‐Analysis of Randomized Trials
Source: Clin Cardiol. 2026 May 12;49(5):e70350. doi: 10.1002/clc.70350 (PMC13162227; doi:10.1002/clc.70350)
Supplement: Supplementary file 1 — Figure S1: Risk of bias graph.Figure S2: Risk of bias summary.Figure S3: Pool analysis of radial artery occlusion (RAO) in included studies comparing dTRA and TRA.Figure S4: Funnel plot for the endpoint of radial artery occlusion (RAO).Figure S5: Pool analysis of hematoma in included studies comparing dTRA and TRA.Figure S6: Pool analysis of arteriovenous fistula (AVF) in included studies comparing dTRA and TRA.Figure S7: Pool analysis of pseudoaneurysm in included studies comparing dTRA and TRA. [file CLC-49-e70350-s001.docx]

Supplementary Table 1. **Design of included studies**

| **Identifier** | **Study ID** | **Study design** | **Inclusion criteria** | **Exclusion criteria** |
| --- | --- | --- | --- | --- |
| NCT04318990 | Al-Azizi K (2024) [16] | Single center  RCT  Open-label | Patients were required to be at least 18 years of age, have palpable and normal blood flow in the distal and proximal radial arteries, be able to comply with the protocol and sign an informed consent form | Patients with specific access requirements, medication history, medical history, participation in other studies, medical conditions, surgical plans, anatomical structure issues, etc |
| NCT04171570 | Aminian A (2022) [17] | Multicenter  RCT  Open-lable | Adult patients aged 18 or older who provide written informed consent and are suitable for dTRA or TRA with the 6-F Glidesheath Slender | Patients with medical conditions that may result in non-compliance/confounded data interpretation, prolonged haemodialysis, ST-segment elevation myocardial infarction, or chronic total occlusion PCI are excluded |
| NCT04211584 | Babunashvili AM (2024) [18] | Multicenter  RCT  Open-lable | Patients older than 18 years of age with chronic or acute coronary syndromes (non-ST-segment elevation myocardial infarction) who require coronary angiography or percutaneous coronary intervention | Patients with previous percutaneous intervention through the same radial artery, coagulation disorders, life expectancy less than 1 year, and artery diameter less than 1.5▒mm |
| NCT05253820 | Chen T (2024) [19] | Single center  RCT | Patients aged ≥18 years with palpable distal and conventional radial arteries who were willing to participate in the study and signed an informed consent form | Cardiogenic shock or acute ST-segment elevation myocardial infarction, age ≥90 years, height ≥185▒cm, previous history of right radial or distal arterial intervention, contraindications to puncture site, and anticipation of missed follow-ups |
| ***Continued on the next page*** | | | | |
| NCT04125992 | Daralammouri Y (2022) [20] | Single center  RCT  Partially blinded | Patients hospitalised for cardiac catheterisation with palpable proximal and distal radial artery pulsations | Patients with non-palpable bilateral radial arterial and venous fistulas, haemodynamic instability, ST-segment elevation myocardial infarction, radial arteriovenous fistulae used for haemodialysis, coronary artery bypass grafting using the left or right radial arteries, etc., as well as patients suffering from Raynaud’s phenomenon, previous occlusion of the radial arteries, incomplete palmar arches and lymphoedema |
| NA | Gupta M (2023) [21] | Single center  RCT  Single blinded | Patients older than 18 years of age with symptoms of coronary artery disease and palpable radial artery in the anatomical snuffbox | Patients with contraindications to contrast, arteriovenous fistulae in the forearm, a history of coronary artery bypass grafting in which the radial artery was used as a graft vessel, and type III and IV radial arteries |
| NA | Kılıç R (2023) [22] | Multicenter  RCT | Consecutive patients from three centres between April 2021 and May 2022 | Acute ST-segment elevation myocardial infarction, cardiogenic shock, haemodynamic instability, use of non-6 French catheters, refusal to participate and age over 75 |
| NCT05982366 | Koziński Ł (2023) [23] | Single center  RCT | Inclusion of adult Caucasian patients scheduled for coronary angiography or percutaneous coronary intervention | ST-segment elevation myocardial infarction, sudden cardiac death, haemodynamic instability, chronic kidney disease (stage 4-5), occlusion of the forearm artery, previous failure of the ipsilateral radial artery route, inappropriate diameter of the radial artery, and unavailability of ultrasonography |
| NA | Sharma AK (2020) [24] | Multicenter  RCT  Open-lable | Patients with chronic stable angina who need coronary angiograhy. | Patients with an arteriovenous fistula in the forearm for haemodialysis, patients using the radial artery as graft material after coronary artery bypass grafting, patients with radial artery types III and IV, and patients who do not wish to undergo this procedure |
| ***Continued on the next page*** | | | | |
| NCT04801901 | Tehrani BN (2024) [25] | Single center  RCT  Open-label | Age ≥18 years, undergoing non-emergency coronary angiography (CAG), percutaneous coronary intervention (PCI), or both, and agreeing to 90-day follow-up | Known pregnancy, uncorrected bleeding disorders, inability to take antiplatelet agents, prior bilateral radial artery puncture, ST-segment elevation myocardial infarction, cardiogenic shock, and allergy to stainless steel, platinum, chromium, nickel, molybdenum or everolimus |
| NCT03986151 | Tsigkas G (2022) [26] | Single center  RCT  Open-label | Patients older than 18 years of age with indications for coronary angiography or percutaneous coronary intervention | Patients with ST-segment elevation myocardial infarction, haemodynamic instability, nonpalpable right radial artery, previous coronary artery bypass grafting, anatomical limitations, and high probability of non-compliance with study protocols |

Supplementary Table 2. **Measures of hand function by grip strength, pinch strength and questionnaires**

| **Study ID** | **Baseline and follow-up** | **Grip Strength (kg)** | **Pinch Strength (kg)** | **DASH** | **Quick DASH** |
| --- | --- | --- | --- | --- | --- |
| Babunashvili AM (2024) [18] | Baseline | TRA: 40.0 (30.0-49.0)  dTRA: 38.0 (28.0-48.0) | TRA: 10.0 (7.0-12.0)  dTRA: 9.5 (7.0-12.5) | NA | NA |
|  | 12 months | TRA: 42.0 (30.0-51.0)  dTRA: 40.0 (28.0-50.0) | TRA: 11.0 (8.5-14.0)  dTRA: 10.0 (30.0-49.0) | NA | NA |
| Al-Azizi K (2024) [16] | Baseline | TRA: 24.0 (17.0-31.3)  dTRA: 26.0 (18.9-36.0) | TRA: 7.1 (5.4-8.2)  dTRA: 7.9 (5.7-9.1) | NA | TRA: 4.6 (0.0, 15.9)  dTRA: 4.6 (0.0, 13.6) |
|  | 12 months | TRA: 25.3 (18.8-31.7)  dTRA: 29.2 (18.0-37.2) | TRA: 6.9 (5.0-8.0)  dTRA: 8.0 (6.0-9.0) | NA | TRA: 4.6 (0.0, 12.0)  dTRA: 2.7 (0.0, 13.6) |
| Tehrani BN (2024) [25] | 3 months | NA | NA | TRA: 8.0▒±▒12.9  dTRA: 5.7▒±▒10.2 | NA |
