# Supplementary material for: Hand Dysfunction After Intervention via Distal Versus Conventional Transradial Access: A Meta‐Analysis of Randomized Trials
Source: Clin Cardiol. 2026 May 12;49(5):e70350. doi: 10.1002/clc.70350 (PMC13162227; doi:10.1002/clc.70350)
Supplement: Supplementary file 2 — Table S1: Design of included studies.Table S2: Measures of hand function by grip strength, pinch strength and questionnaires. [file CLC-49-e70350-s002.docx]

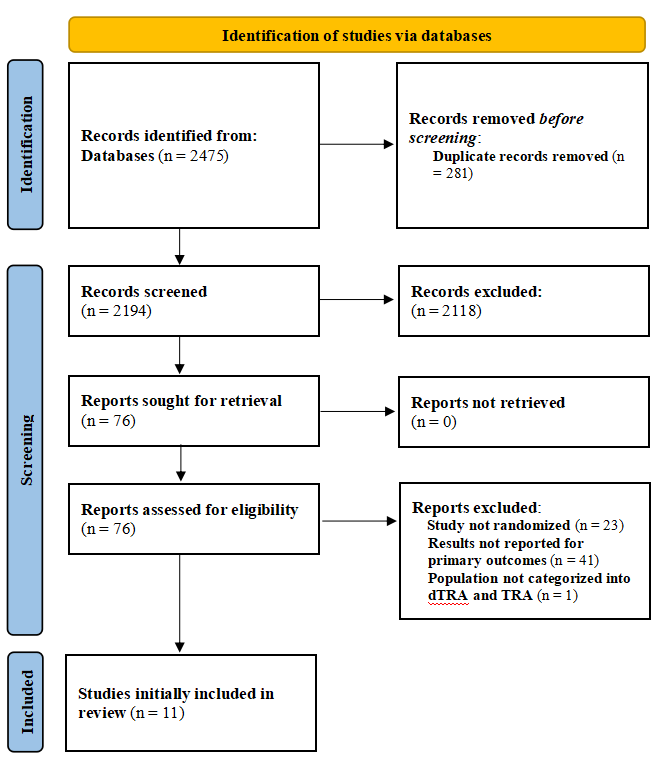


**Figure 1**. Flow diagram of the search for studies included in the Meta analysis according to the Preferred Reporting Items for Systematic Reviews and Meta-Analyses (PRISMA)


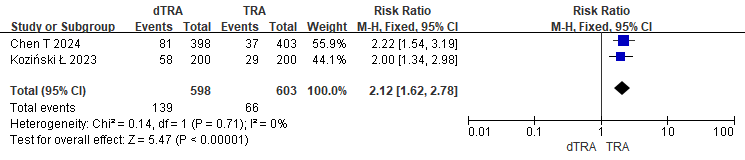


**Figure 2**. Main analysis of hand numbness in included studies comparing dTRA and TRA (Mantel-Haenszel fixed-effect)


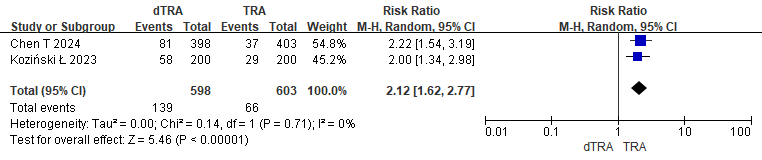


**Figure 3**. Sensitivity analysis of hand numbness in included studies comparing dTRA and TRA (Mantel-Haenszel random-effects)


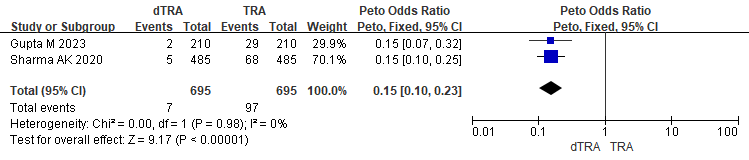


**Figure 4**. Main analysis of hand persistent pain in included studies comparing dTRA and TRA (Peto fixed-effect)


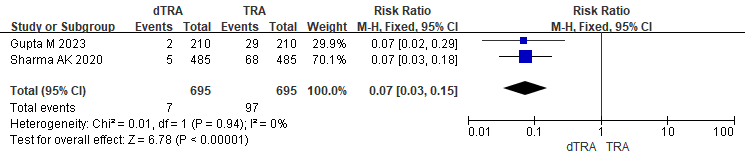


**Figure 5**. Sensitivity analysis of hand persistent pain in included studies comparing dTRA and TRA (Mantel-Haenszel fixed-effect)


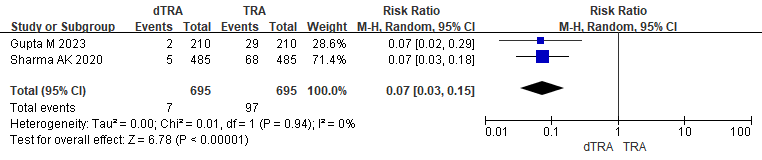


**Figure 6**. Sensitivity analysis of hand persistent pain in included studies comparing dTRA and TRA (Mantel-Haenszel random-effects)


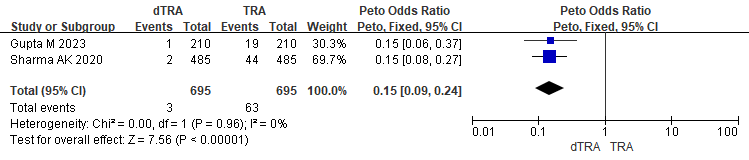


**Figure 7**. Main analysis of hand clumsiness in included studies comparing dTRA and TRA (Peto fixed-effect)


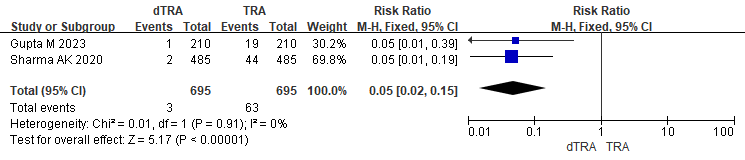


**Figure 8**. Sensitivity analysis of hand clumsiness in included studies comparing dTRA and TRA (Mantel-Haenszel fixed-effect)


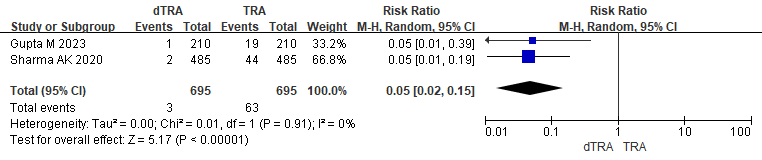


**Figure 9**. Sensitivity analysis of hand clumsiness in included studies comparing dTRA and TRA (Mantel-Haenszel random-effects)


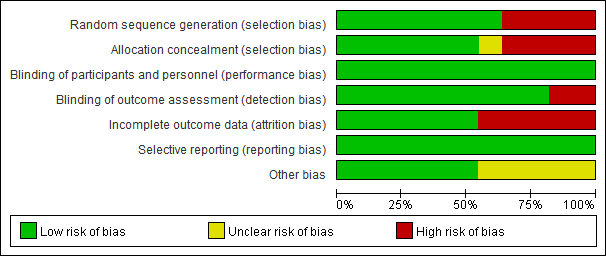


**Supplementary Figure 1**. Risk of bias graph


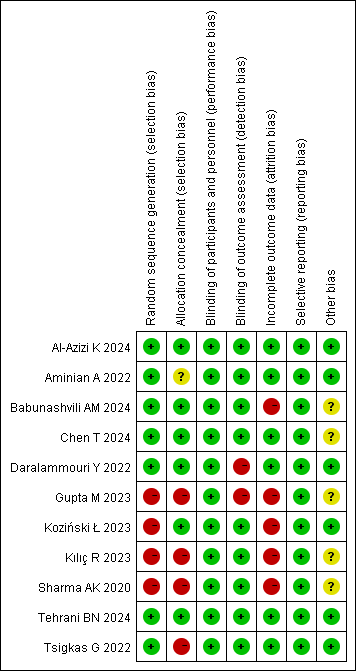


**Supplement Figure 2**. Risk of bias summary


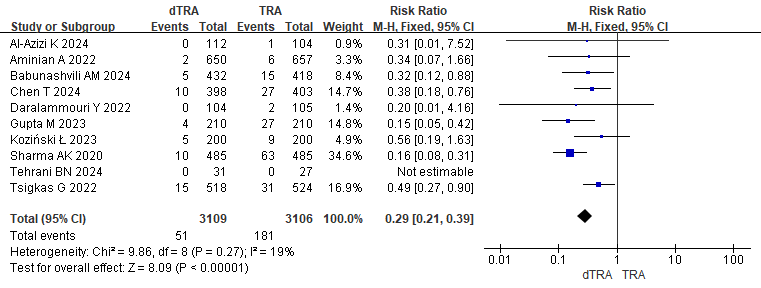


**Supplement Figure 3**. Pool analysis of radial artery occlusion (RAO) in included studies comparing dTRA and TRA


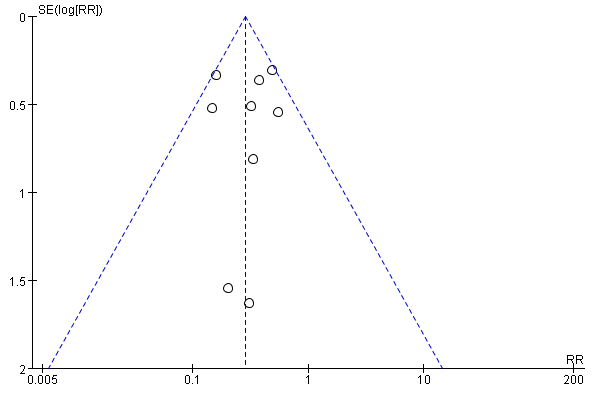


**Supplement Figure 4**. Funnel plot for the endpoint of radial artery occlusion (RAO)


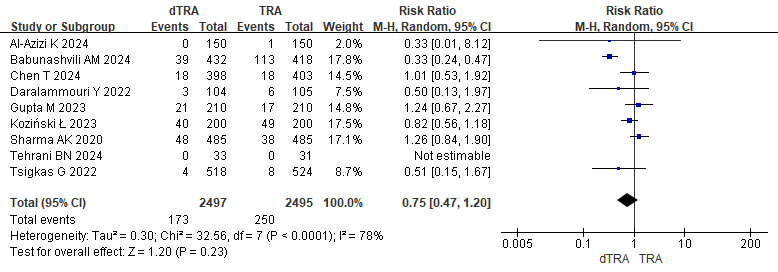


**Supplement Figure 5**. Pool analysis of hematoma in included studies comparing dTRA and TRA


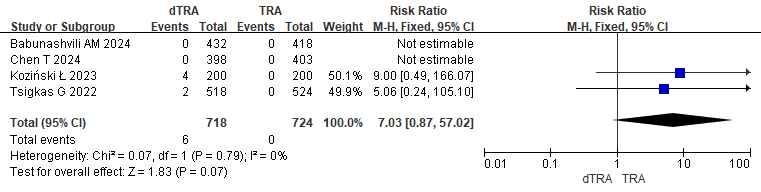


**Supplement Figure 6**. Pool analysis of arteriovenous fistula (AVF) in included studies comparing dTRA and TRA


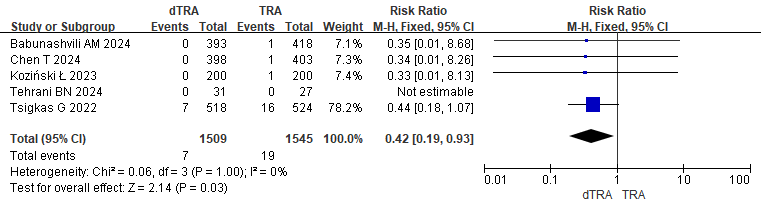


**Supplement Figure 7**. Pool analysis of pseudoaneurysm in included studies comparing dTRA and TRA
